# The status of neurology fellowships in the United States: clinical needs, educational barriers, and future outlooks

**DOI:** 10.1186/s12909-021-02536-8

**Published:** 2021-02-17

**Authors:** Harini Sarva, Gustavo A. Patino, Mehmood Rashid, James W. M. Owens, Matthew S. Robbins, Stefano Sandrone

**Affiliations:** 1 A.B. Baker Section on Neurological Education, American Academy of Neurology, Minneapolis, USA; 2 Weill Cornell Medicine, New York, NY USA; 3 Oakland University William Beaumont School of Medicine, Rochester, MI USA; 4 The University of Toledo, Toledo, OH USA; 5 University of Washington, Seattle, Washington USA; 6 Imperial College London, London, UK

**Keywords:** Neurology fellowship, Medical education, Neurologic education, Factors, Choice, Curriculum design

## Abstract

The need for subspecialty-trained neurologists is growing in parallel with increasing disease burden. However, despite the immense burden of neurological diseases, like headache and neurodegenerative disorders, recruitment into these subspecialties remains insufficient in the United States. In this manuscript, a group of educators from the American Academy of Neurology’s A.B. Baker Section on Neurological Education sought to review and discuss the current landscape of neurology fellowships in the United States, the factors driving fellowship recruitment and the educational barriers. Moreover, suggestions to potentially improve recruitment for under-selected fellowships, which can contribute towards an alignment between neurological education and neurological needs, and future educational scenarios are discussed.

## Background

With the burden of neurological disorders sharply rising, there is a pressing need for subspecialty neurology training in the USA [1]. The Global Burden of Disease collaborators report that migraine, Alzheimer’s disease (AD) and other dementias, and Parkinson’s disease (PD) ranked second, third, and eleventh in terms of disease-adjusted life-years (DALYs, which is the sum of years of life lost and years lived with disability) by age and sex, among the fifteen neurological conditions causing high DALYs worldwide. In 2016, US prevalence estimates for migraine, AD, and PD were 47,016,985, 4,029,450, and 707,158, respectively [2–4]. Despite this, the National Center for Health Workforce in 2017 predicted that between 2013 and 2025 the supply of neurologists will likely grow by 11%, while demand will grow by 16%, resulting in an 820-physician full-time equivalent (FTE) shortfall [5, 6]. For headache specifically, a recent workforce gap analysis demonstrates that, despite only around 500 certified headache specialists in the USA, 3700 specialists are needed to care for the most affected people [7]. In a 2015 survey of graduating neurology residents, only 6.6 and 5.9% of respondents chose Headache Medicine and Cognitive Disorders as potential fellowships (with only 2.8% of respondents not pursuing any fellowship training) [8]. There is a mismatch between the DALY’s of these conditions and numbers of trained subspecialty providers. This is influenced not only by the deficiency of the neurology workforce, but also by the subsequent subspecialty choices of those training in neurology.

## Main text

A group of educators from the American Academy of Neurology (AAN) A.B. Baker Neurological Education Section reviewed the current status of neurological fellowships through published literature in PUBMED and from governing bodies such as the Accreditation Council for Graduate Medical Education (ACGME) and the United Council for Neurologic Subspecialties (UCNS). Factors influencing fellowship choice have been discussed, and recommendations to improve recruitment for under-represented subspecialties have been proposed. As this is a narrative literature review, without study subjects, no IRB application was submitted.

### The current landscape of clinical neurology fellowships in the US

There are currently 31 types of neurology subspecialty fellowships (see Tables 1 and 2) according to the AAN, ACGME, and UCNS with 17 of these subspecialties accredited by either ACGME or UCNS. Residents apply to fellowships through three means depending on the subspecialty. The Electronic Residency Application Service (ERAS) handles applications to: Brain Injury Medicine, Sleep Medicine, Interventional Radiology, Vascular Neurology, Neuroradiology, and Pain Medicine [12]. The San Francisco (SF) Match handles applications for: Movement Disorders, Neurocritical Care, Neuro-Oncology, and Neuro-Otology [13]. Other subspecialty fields utilize the direct application to programs themselves (e.g., headache medicine, neurophysiology, and epilepsy). The use of multiple match processes in a medical specialty is not unusual; anesthesiology, surgery, and orthopaedics also have fellowship matches in both National Resident matching program (NRMP) and the SF Match. This process likely reflects business decisions by the subspecialty governing bodies and which match organization can better address their particular needs.

**Table 1:** Accredited and non-accredited Neurology Fellowships

Accredited Fellowships	Accrediting Body	Number of Sites in the US
Brain Injury Medicine [9]	ACGME	1
Clinical Neurophysiology [9]	ACGME	89
Endovascular Surgical Neuroradiology [9]	ACGME	2
Epilepsy [9]	ACGME	77
Neuromuscular Medicine [9]	ACGME	49
Pain Medicine [9]	ACGME	104
Sleep Medicine [9]	ACGME	84
Vascular Neurology [9]	ACGME	99
Autonomic Disorders [10]	UCNS	5
Behavioral Neurology and Neuropsychiatry [10]	UCNS	35
Clinical Neuromuscular Pathology [10]	UCNS	5
Headache Medicine [10]	UCNS	41
Geriatric Neurology [10]	UCNS	4
Neural Repair and Rehabilitation [10]	UCNS	0
Neuro-oncology [10]	UCNS	34
Neurocritical Care [9, 10]	UCNS; ACGME	70
Neuroimaging/Neuro-radiology [10]	UCNS	5

**Table 2:** Accredited and non-accredited Neurology Fellowships. Data from unfilled positions are not publicly available

Non-Accredited Fellowships	Number of Sites in the US [11] for all but movement disorders [12]
Balance Disorders	0 [11]
Clinical Research	2 [11]
Cognitive Disorders	1 [11]
Complimentary Medicine	0 [11]
Movement Disorders	48 [12]
Neuro-Ophthalmology	1 [11]
Neuro-Otology	0 [11]
Neuroendocrinology	0 [11]
Neurogenetics	0 [11]
Neurohospitalist	2 [11]
Neuroimmunology/multiple sclerosis	20 [11]
Neuropharmacology	0 [11]
Other (Infectious disease, intraoperative monitoring, Therapeutic Development)	8 [11]

The ACGME is a non-profit organization which accredited 830 institutions, 11,200 residency programs, and 180 fellowships in 2018 [14]. Accreditation occurs through a voluntary review and evaluation process to ensure that the training programs meet established quality standards. In addition to accreditation, the ACGME provides recognition for institutions and/or programs based on a voluntary evaluation process [14].

The UCNS is a non-profit organization that accredits smaller neurology fellowships and provides certification for fellows. It is governed by a board of neurologists from parent organizations, such as the AAN and accredited subspecialties [15]. For both the ACGME and the UCNS, leaders in subspecialty fields develop competency standards.

### Key factors behind the choice of a subspecialty fellowship

Several factors influence fellowship choices, including recommendations or modelling by educational leaders, the diversity of neurology subspecialty exposure and elective opportunities within training, opportunities in research and clinical practice, and considerations related to the work-life balance.

### Subspecialty preferences of educational leaders

Trainees may be inspired by role models in their institutions, including chairpersons and residency program directors (PDs). These faculty drive curricular organization and priorities that may be biased towards their own particular subspecialties. Though the ACGME lists subspecialties as “aspects of neurology” requiring didactics, faculty expertise, and clinical exposure [16], their curricular weight is left to individual training programs and subjected to the interests of departmental education leaders. Moreover, some aspects of neurology are not specifically represented in ACGME program requirements, including headache, which is the most common reason for neurological consultation [17, 18].

A study analysed the subspecialty training undertaken by 72 adult neurology chairpersons and PDs [19]. Given that chairpersons are generally older than PDs and more likely to have trained in an era of less well-developed fellowships, it was unsurprising that 27.3% of chairpersons reported no fellowship, compared to only 13.9% of PDs. Clinical neurophysiology was the most commonly reported fellowship (32.6% of chairpersons and PDs), with a close divide between electroencephalography/epilepsy and electromyography/neuromuscular tracks. Movement disorders training demonstrated the biggest difference between chairs (4.5%) and PDs (10.2%), perhaps owing to more recent fellowship organization, with a similar relative, but smaller absolute, increase in headache fellowship training (0.8% vs 3.6%). However, outpatient subspecialty areas, including dementia/behavioral neurology, headache, movement disorders, and sleep, were generally underrepresented.

Demographics, such as gender of neurology leaders, may be an important factor behind subspecialty choice and the decision to pursue either an academic or non-academic career. Given that only 14.4% of chairpersons and 32.1% of PDs are women [19], there may be a paucity of role models for women interested in pursuing subspecialty careers.

### Neurology graduate medical education exposure

Experiences during neurology residency are critical in crystallizing subspecialty fellowship interests, but these experiences are typically weighted towards inpatient disciplines, particularly early in training. Given the lack of a single timeline for fellowship applications [20] and application deadlines as early as the end of the PGY2 year, programs prioritizing earlier subspecialty exposure may be better positioned to aid in fellowship choices. Since 2007, the proportion of categorical neurology residency programs has increased, reaching 59% in 2016 [21]. Structurally, such categorical programs are well-positioned to feature neurology rotations, including electives, during the PGY1 year. Those early experiences could either include underrepresented subspecialties or make time available later in the curriculum for them [21]. However, the degree to which this is taking place or how this organization impacts fellowship choice is currently unknown.

Curricular design is significantly influenced by the ACGME mandated milestones [22], which is a developmentally organized progression of expectations for residents managed by ACGME competency domains. Though subspecialty domains are generally covered, there are aspects of neurology that are not, as neurointensive care and pain medicine. While not mandated by milestones, some subspecialty areas such as headache and neurocritical care are assessed by the neurology in-service exam, which is another influence on curricular design. As ever-increasing varieties of content areas emerge as relevant to residency training, a flipped-classroom model may be a strategy to ensure a more balanced delivery of subspecialty educational content [23, 24].

Another potential area limiting neurology resident exposure is faculty staffing of inpatient and outpatient services. In an era witnessing an increase in the use of neuro-hospitalists [25], subspecialists primarily in ambulatory neurology may not rotate on such services and, therefore, residents may not routinely encounter these faculty during inpatient rotations. Though patient care may be more streamlined and effective in a neuro-hospitalist model [26], this framework places subspecialists at risk of existing in ‘silos’ and not in general neurology education settings, where more consistent trainee interactions occur. Earlier and broader exposure to outpatient neurology would serve to counter this trend.

### Do experiences in medical school contribute to fellowship choice?

The decision to pursue neurology as a career often emerges during medical school clerkships. Yet, such undergraduate clinical experiences typically focus on inpatient exposure which further disadvantages fellowships grounded in ambulatory neurology. A recent neurology core curriculum guideline by the AAN Undergraduate Education Subcommittee and Consortium of Neurology Clerkship Directors suggested a good balance among outpatient and inpatient neurology topics [27]. However, their weighting is still left to the individual institutional preference and expertise.

Neurologists are underrepresented in key medical school leadership positions such as deans [28]. This can potentially limit exposure of students to the significant diversity of neurology through lack of preclinical neurologic physical diagnosis courses, introductory clinical neurology opportunities such as lumbar puncture simulations, or creation of subspecialty electives in clinical years.

### Access to national resident education programs

Subspecialty societies have developed several national subspecialty resident education programs. Some programs, including the American Headache Society Resident Education Program, the Movement Disorders School for Neurology Residents, and the J. Kiffin Penry Residents Epilepsy Program, explicitly target first- or second-year residents to influence fellowship choice. Though residents earlier in their training may not have much schedule flexibility to travel, education programs that represent disciplines featuring an insufficient number of fellows or specialists may consider shifting eligibility criteria to residents undifferentiated in a subspecialty choice or provide virtual options to increase participation. Besides, the lack of awareness of these programs, probably due to an inadequate advertisement by residencies, further compound the problem of reduced early exposure.

### ‘Hands-on’ features of clinical neurology practice

The incorporation of procedures may enhance the appeal to trainees of particular subspecialties. Though practice patterns demonstrate increased procedures related to headache and movement disorders, such as botulinum toxin injections, deep brain stimulation programming, and nerve blocks [29], more recent advances in acute stroke care may potentially draw more trainees to dedicated interventional neuroradiology fellowships [30, 31]. Clinical neurophysiology on an electromyography track was a top choice among neurology graduates [19]. Still, substantial changes in Medicare reimbursement in 2013 led to reductions in resource utilization of nerve conduction studies, potentially dissuading trainees from pursuing this subspecialty [32]. The rapid growth of interest in neurocritical care may also highlight the importance of procedures to at least some trainees.

### Research and fellowship funding

Funding opportunities for research may influence trainees’ pursuit of academic careers. The National Institute of Health provides federal funding for career development awards for early stage investigators. However, budget allocation does not always match the disease burden of many neurological subspecialties [33]. In addition to this disparity, funding of research and clinical fellowships, regardless of ACGME accreditation, may be inconsistent and lead to an inability to develop long-term fellowships. Inconsistent financing sources include foundations, industry, and philanthropy. This issue seems particularly relevant for fellowships without ACGME accreditation [34], such as movement disorders, headache, and multiple sclerosis fellowships.

### Burnout

Burnout in neurology is a major issue that may influence subspecialty fellowship choices [35]. Nearly three-quarters of all neurology residents have at least one symptom of burnout [36]. In the large AAN burnout survey study, only subspecialists in epilepsy had significantly fewer burnout symptoms than those practicing general neurology [35]. Neuro-hospitalist medicine, neurointensive care, and interventional neurology may provide a desirable career option because of a clinical shift-work structure akin to hospitalists in internal medicine (IM). Interestingly, IM residency PDs report lower burnout rates than IM physicians and medical education administrative leaders overall [37]. It is unknown if this discrepancy also applies to neurology PDs, but, if so, may elevate further the importance of subspecialty choices of PDs and modelling by trainees.

### Barriers to choosing neurology fellowships

**Fig. 1:**
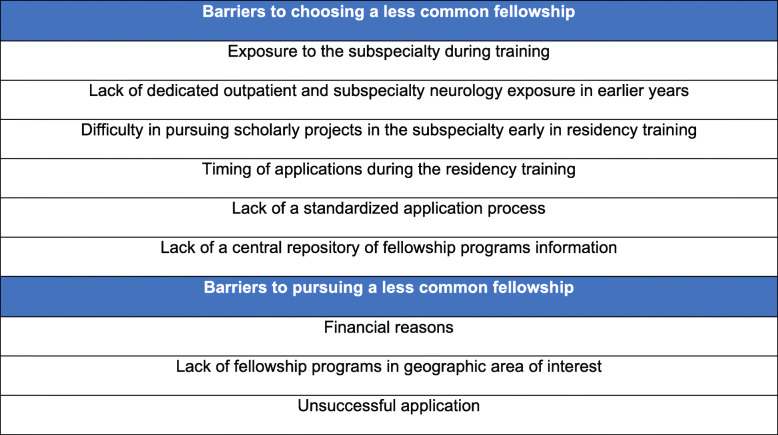
Barriers to choosing and pursuing less common fellowship training

While the majority of US neurology residents pursue fellowships (90% in the 2017 survey of graduating residents) [38], there are still multiple barriers to either accessing advanced training or fully exploring all available options (Fig. 1). In the 2015 Fellowship Survey Report [8], the 805 participants included PGY3 and PGY4 residents, current fellows, and specialists who completed fellowship within the previous 2 years. Out of 499 respondents who were residents, only 16 (3.2%) were not planning to pursue a fellowship. While at least half of these indicated that they preferred general neurology and felt prepared to enter the workforce, 6 (37.5%) listed financial reasons, including the level of educational debt and family obligations [8]. The data for the influence of debt on medical student specialty choice seems mixed. On the one side, even though this consideration influenced only 1% of all surveyed residents, the scale of the problem is potentially considerable. In fact, the 2017 survey of graduating neurology residents listed the median reported debt at $180,000 (interquartile range of $80,000 to $250,000), and 32% of respondents had debt equal or greater than $250,000 [39]. On the other side, as an alternative explanation, residents might want to get into highly remunerative subspecialties (i.e., neurointervention, neurocritical care) rather than less remunerative subspecialties like headache. The 2019 Executive Summary of the AAN Neurology Compensation and Productivity Report listed the average annual compensation for general neurology ($251,385.10) in the 68th percentile of compensation for neurological subspecialties (range: $153,549.70- $545,729.90; interquartile range: $212,586.72–$258,135.37), and above 13 subspecialties [40]. Given this landscape, it is reasonable that, at least for some graduates, not all the fellowships would have the same return on investment in terms of deferred earnings. Other reasons for not considering fellowship included having accepted job offers (25%), lack of fellowship programs in the preferred geographic area (6.5%), and going into research (6.5%) [8].

As mentioned above, the main barrier reported was the lack of specific subspecialty exposure during residency. Respondents pursuing a fellowship in the 2015 Fellowship Survey Report ranked clinical, personal, and research interests as the top three factors in choosing a fellowship [8]. The next top factor was an influential role model or mentor. Complicating this lack of exposure is the early fellowship application deadline, taking place as early as two years ahead of graduation [8].

According to the 2017 survey of graduating residents, the lack of exposure before deciding on fellowships influenced 46% of adult neurology and 14% of child neurology residents [38]. The concern for the timing of fellowship application is shared by 54% of graduating residents [38] and 78% of PDs, with multiple participants in both surveys advocating that the application period be moved to the end of PGY3 or early PGY4 year [20]. A better distribution of neurology elective time across the 4 years of residency, rather than back-loading elective time towards the end when trainees have already applied for fellowships, may be a good strategy to mitigate the effect of the prematurely early fellowship application timelines [20]. In February of 2020, a consortium including the AAN, the UCNS, the American Neurological Association, and other associations and societies involved in neurological care education, penned a position statement recommending the adoption of a universal timeline for fellowship recruitment. The recommendation was to begin the fellowship application process in March of the PGY-3 year, with fellowship offers made no earlier than August of the PGY-4 year [9]. The AAN is leading the engagement of leadership and programs from the different neurological subspecialties to facilitate the implementation of this universal timeline [9].

The lack of standardized fellowship application process is another barrier. Many fellowships do not participate in the ERAS or SF Match, utilizing direct application processes [39]. Furthermore, in these direct processes, there is no general standard for the application requirements, and each program can have unique demands [39], making it more difficult for residents to be prepared the application materials. The AAN is leading an effort to provide a centralized source of information about the different programs, but the 2015 Fellowship Survey Report still includes multiple comments expressing the desire for a more centralized process [8].

Finally, while mixed sources of funding have made fellowships historically less dependent on federal funding than residencies [10, 11], macroeconomic issues have the potential to disrupt a pipeline that is already struggling to meet the demand. This is particularly important when political proposals for reforming the health care system might change the *status quo* of practice.

## Discussion

Understanding prospectively what trainees in their first years of residency know about subspecialties can aid in developing curricula or early elective opportunities to ensure a broader exposure. Participation in innovative therapies, procedures, and clinical trials for subspecialties such as headache and movement disorders may spur an interest in choosing either of these fields.

Improving outpatient clinic experiences can advance recruitment into ambulatory subspecialties. Greater outpatient contact time alone may not be enough, and training in inefficient outpatient settings may negatively impact residents’ perception of mostly outpatient subspecialties such as headache medicine. Better organization of patient appointments and more continuity of care in subspecialty clinics will allow them a better opportunity to understand outpatient neurology. Though the neurology residency review committee of the ACGME recently instituted a new requirement for residents to attend 40 continuity clinics in each of their PGY2, PGY3, and PGY4 years, there is neither a PGY1 requirement for outpatient experiences nor a mandate for any specific outpatient subspecialty exposure [41]. However, having subspecialty clinics alone is insufficient for improving exposure. Providing residents more regularly scheduled subspecialty clinics, having the same patients scheduled with a given resident, and pairing residents with the appropriate subspecialty attending can improve understanding of chronic management of these challenging patients. This will also allow for siloed subspecialists to have more significant interaction with and the potential to mentor residents. Lessons should be learned from training programs in other specialties with an outpatient bias, including family medicine and the primary care track of internal medicine. Neurology residency PDs face a difficult choice when deciding between increasing resident contact with outpatient neurology and maintaining the quality of resident education during inpatient rotations. One innovative consideration would be to combine separate inpatient rotations, such as neurocritical care and vascular neurology, to reduce time inefficiency while maintaining exposure. Since most patients in the neuro-ICU will also be cared for by the vascular neurology and/or neurosurgery team, residents can interact with and learn from such teams during the same rotation. Many of today’s medical students are or will be exposed to varying degrees of a longitudinally integrated clinical curriculum (LIC) in medical school, and residency programs may benefit from applying similar educational philosophy. The ‘curricular crush’ is a concern throughout the whole spectrum of medical education. Given the remarkable disparity in program designs, it is difficult to make specific recommendations. Yet, overall, the PGY-1 period may be an intriguing opportunity to provide early exposure to underrepresented subspecialties, especially those that are more outpatient-based. Between 2007 to 2016, the percentage of categorical neurology residency programs has increased from 30 to 59%, thus providing an opportunity to include longitudinal and subspecialty clinic experiences for these PGY-1 trainees [21].

Ongoing efforts by the AAN and other leading bodies in neurology practice and education to standardize the fellowship application process taking place no earlier than the end of the PGY-3 year are significant developments. Such change would improve resident exposure to more subspecialties before they have to commit to a fellowship, not only in terms of patient access, but also in terms of participation in research and networking.

Incorporating residents into divisional research meetings or conferences by inviting them throughout the year can also increase exposure. While it is challenging to attend every session, receiving notifications about the topics may inspire them to attend, or at least be aware of the interesting concepts and research being discussed. Subspecialty divisions may also consider hosting regional conferences in collaboration with institutions that are in geographic proximity, in-person or virtually. This might give residents a chance to present their work, meet fellowship directors from other institutions, and collaborate with residents and fellows from these institutions. It can also be advantageous for smaller residency programs without a fellowship program or adequate clinical representation of a certain subspecialty locally. Participation in educational activities hosted by state neurological societies may provide similar opportunities.

On a faculty level, with increased financial pressures and less reimbursement for education [42], it is imperative to engage residents from the beginning, particularly if they come with requests for research opportunities or participation in interesting case discussions requiring subspecialty expertise. The movement for more categorical neurology residency program positions may help to feature early clinical and scholarly exposure in such subspecialties. Following up with trainees on projects or cases may help in closing the loop and keeping them engaged and interested in subspecialty neurology.

However, it may be that these interventions occur too late, especially if graduating medical students rank specific training programs based on the strengths of particular subspecialties to which they have been exposed to during their clerkships and electives. As many medical schools revamp their foundational science curriculum to include more clinical content, opportunities exist for subspecialty neurologists to teach junior medical students and to start forming their opinions regarding subspecialties even in preclinical years. The ‘pipeline’ does not need to end with medical students choosing to do a neurology residency. Programs that are now seeing medical students on the new curriculum applying to residency, or starting residency, can seek data regarding what part of the preclinical experience helped and what hindered recruitment into neurology and certain subspecialties. This is a population that can be followed longitudinally to gain more insight into this topic.

Finally, the optimal accreditation strategy for neurology fellowships still has to be defined. At the least, all fellowship disciplines should have accreditation to standardize educational experiences, share resources, and offer legitimization. Accreditation may be dynamic, as exemplified by neurocritical care, a UCNS accredited subspecialty, recently achieving ACGME credentialing status. However, a singular unifying body for all fellowships may help in many ways. For example, it can be instrumental in aligning different application timelines, promote protected time for fellowship PDs, advocate for program funding. It can also help leading national efforts to match the demand of patients with neurological disorders to the physicians needing to advance their training to better care for and study them, thus aligning neurological education with care needs.

We can learn from other specialities that have a significant outgrowth of fellowships. For example, the American Medical Association has provided trainees access to FREIDA™, a comprehensive tool that allows trainees to search for information about 11,000 training programs which are ACGME accredited. This offers trainees the opportunity to obtain information regarding location, benefits, special tracks, and other features of the program. This single-source also has status updates on which programs are currently accepting applications and how to apply, either through ERAS or through direct application to the program. The AAMC through ERAS provides further direction on applying to the various subspecialties across several disciplines, making it a singular application process [43]. Having access to a single source that provides updated and evolving information about various fellowships is invaluable for trainees.

There are some limitations to our work. For example, no recent national resident survey data exist regarding fellowship choice after initiation of newer ACGME outpatient requirements, and there is a scarcity of information on other programs’ efforts across the USA to improve subspecialty fellowship placement. In terms of future research avenues, larger educational studies to open dialogue and make appropriate changes at different levels are warranted.

## Conclusions

**Fig. 2:**
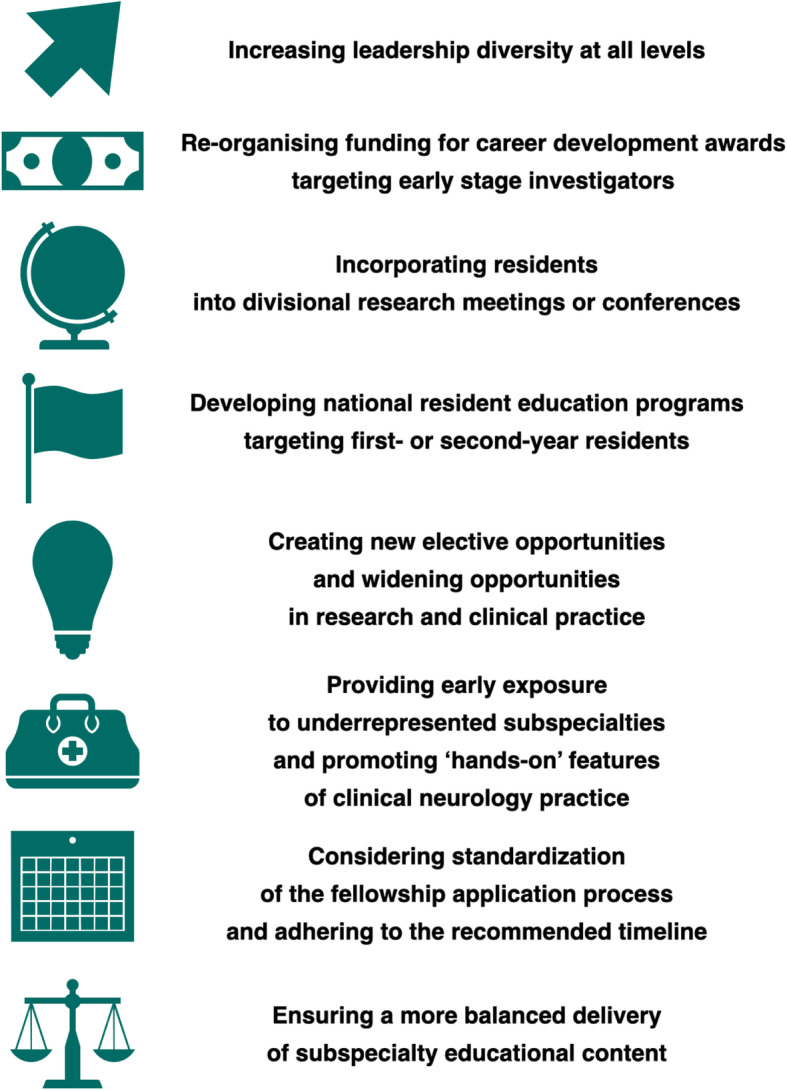
Key recommendations

There is a mismatch between subspecialty expertise and clinical care needs in the US, which contributes to inadequate care of neurological disorders, including migraine, AD, and PD. Several barriers remain, but these can be addressed through interventions designed to expose and mentor residents and medical students early on to foster interest in underrepresented subspecialties (Fig. 2). Though substantial efforts exist to provide residents with subspecialty exposure and mentorship, there is much to be done to enhance recruitment into specific fields, such as headache medicine and behavioral neurology, to compensate for the lack of subspecialty care for these chronic patients.

## Data Availability

Not applicable.
